# A lightweight and robust method for electrocardiogram anomaly detection and localization using multi-scale masked autoencoder

**DOI:** 10.1371/journal.pone.0343571

**Published:** 2026-03-17

**Authors:** Ya Zhou, Yujie Yang, Jianhuang Gan, Xiangjie Li, Jing Yuan, Wei Zhao

**Affiliations:** 1 Department of Information Center, Fuwai Hospital, Chinese Academy of Medical Sciences and Peking Union Medical College, Beijing, China; 2 National Clinical Research Center for Cardiovascular Diseases, Fuwai Hospital, Chinese Academy of Medical Sciences and Peking Union Medical College, National Center for Cardiovascular Diseases, Beijing, China; 3 Center for Health Statistics and Information, National Health Commission of the People’s Republic of China, Beijing, China; Duy Tan University: Dai Hoc Duy Tan, VIET NAM

## Abstract

Electrocardiogram (ECG) analysis is crucial for diagnosing cardiovascular conditions. While traditional classification models require large volumes of labeled data across multiple disease categories, anomaly detection offers a flexible alternative by identifying deviations from normal patterns—an approach particularly valuable given the rarity and diversity of cardiac conditions. However, existing anomaly detection methods often rely on R-peak detection or heartbeat segmentation, which increases preprocessing complexity and reduces robustness to signal variability. To address these limitations, we propose MMAE-ECG, a multi-scale masked autoencoder designed to capture both global and local dependencies without such preprocessing steps. MMAE-ECG integrates a multi-scale masking strategy and a multi-scale attention mechanism with distinct positional embeddings, enabling a lightweight Transformer encoder to efficiently model ECG signals. Additionally, an aggregation strategy is introduced to improve anomaly score estimation. Experiments demonstrate that MMAE-ECG achieves state-of-the-art performance in both anomaly detection and localization while significantly reducing computational costs. Specifically, it requires only approximately 1/78 of the inference FLOPs and 1/18 of the trainable parameters compared to the previous leading method. Ablation studies further validate the contributions of each component, demonstrating the potential of multi-scale masked autoencoders as an effective and efficient approach for ECG anomaly detection.

## 1 Introduction

Electrocardiogram (ECG) is a cost-effective and non-invasive tool widely used in diagnosis of cardiovascular diseases [1–3]. In recent years, deep learning-based ECG classification has made significant progress [4–7], particularly with traditional supervised learning approaches that depend on large, labeled datasets for model training [8]. However, these methods face significant challenges in clinical practice. The high cost of acquiring abnormal ECG data and the limitations of multi-label classification in detecting all possible anomalies—due to restricted label coverage—pose substantial barriers to their widespread application.

Unlike traditional classification methods, which are limited by predefined categories, anomaly detection algorithms offer the potential to identify all abnormal ECG signals. One of the key advantages of anomaly detection methods is that they can be designed to train solely on normal ECG data, effectively bypassing the high cost associated with acquiring rare and diverse abnormal ECG data, a challenge that is often driven by the low prevalence of certain heart diseases [9]. Among the various anomaly detection techniques, generative models have been widely adopted for ECG anomaly detection due to their ability to learn the distribution of normal signals and identify deviations from this learned pattern. For instance, Generative Adversarial Network(GAN)-based methods identify anomalies by measuring the discrepancy between input ECG signals and those generated by the model [10,11]. A representative example is BeatGAN [12], which excels at capturing local beat-level characteristics, making it particularly effective at detecting subtle, localized abnormalities in ECG signals. This localized focus highlights the importance of fine-grained analysis, which is valuable in detecting anomalies at the level of heartbeats.

Nevertheless, ECG signals exhibit significant inter-individual and intra-sample variability, with anomalies manifesting across both global and local temporal scales [9,12]. This variability underscores the need for models capable of capturing multi-scale representations. Recent research has explored the combination of both local and global features, and multi-scale frameworks have shown remarkable promise, achieving state-of-the-art performance in both ECG anomaly detection and localization [9]. In this context, local features refer to representations derived from short segments of ECG signals (e.g., individual heartbeats), while global features capture patterns across the entire signal [13]. The term multi-scale thus denotes the joint consideration of both local and global representations. These advancements underscore the importance of leveraging both fine-grained, localized information and broader, global patterns to improve the accuracy and robustness of ECG analysis

A key approach to achieving such multi-scale representations is the use of mask-based self-supervised learning (SSL) algorithms, which have proven effective in representation learning. These methods can be broadly categorized into two types: one that replaces portions of the input with special tokens, as seen in BERT [14], a strategy also applied in [9]; and another that removes parts of the input and reconstructs the data from the remaining visible portion, as in MAE [15]. Compared to BERT-style methods, MAE-based approaches are more straightforward and computationally efficient, having achieved state-of-the-art performance in visual tasks [16–19]. However, when applied to anomaly detection, MAE often struggles to effectively capture multi-scale features, which limits its overall performance [20]. Despite this, recent studies have shown that MAE-style models, when specifically tailored for ECG analysis, demonstrate notable strengths in capturing morphological patterns, yielding promising results [21–24]. These findings indicate that while MAE excels at modeling certain ECG features, there remains significant potential for improvement, particularly in its ability to capture both local and global multi-scale representations. Thus, enhancing MAE to address these challenges presents a highly promising direction for future research in ECG anomaly detection.

Moreover, clinical environments typically demand models with fast computation and robust performance. A recent approach integrated time series and time-frequency aspects of the ECG signal, reducing parameters and improving computational speed, but it lacks anomaly localization ability [25]. Meanwhile, current methods largely rely on heartbeat segmentation and R-wave detection, making the models highly sensitive to noise and irregularities. Therefore, there is a need for models that are simpler, more efficient and more robust, while being capable of capturing both local and global features.

To address these challenges, we propose a novel multi-scale MAE framework for ECG anomaly detection, referred to as MMAE-ECG, which eliminates the need for R-peak detection or heartbeat segmentation. Our approach leverages a Transformer-based encoder-decoder architecture that integrates a novel multi-scale masking strategy, a multi-scale attention mechanism, and distinct positional embeddings to effectively capture both local and global dependencies in ECG signals. Additionally, an aggregation strategy is employed during inference to refine model predictions. Evaluations on the PTB-XL anomaly detection and localization benchmark demonstrate that MMAE-ECG not only achieves state-of-the-art performance but also significantly improves computational efficiency. Ablation studies further validate the effectiveness of key components, including multi-scale representation learning, local positional embeddings, multi-scale masking, and the aggregation strategy during inference.

The contributions of this work are summarized as follows:

- We propose a novel end-to-end multi-scale Transformer-based framework, MMAE-ECG, for ECG anomaly detection and localization. To our knowledge, this is the first approach that achieves both tasks without relying on R-peak detection or heartbeat segmentation.
- We introduce a multi-scale masking strategy combined with a multi-scale attention mechanism and distinct positional embeddings, enabling the model to effectively capture both global and local dependencies in ECG signals.
- Experiments show that MMAE-ECG achieves state-of-the-art performance while significantly reducing computational costs. Specifically, it requires only approximately 1/78 of the floating-point operations (FLOPs) for inference and approximately 1/18 of the trainable parameters compared to the current leading method.
- Ablation studies validate the effectiveness of key components, including multi-scale representation learning, local positional embeddings, multi-scale masking, and the aggregation strategy during inference.

The remainder of the paper is organized as follows: Section II reviews related work in the field of anomaly detection and localization for time-series data. Section III presents the proposed method in detail, followed by the experimental setup and results in Section IV. Section V discusses the results, and Section VI concludes the paper, highlighting potential avenues for future research.

## 2 Related work

### 2.1 Anomaly detection in time series

Anomaly detection in time series data has attracted significant attention in recent years due to its diverse applications in domains such as economics, manufacturing, and healthcare [26]. Existing methods for anomaly detection can be broadly categorized into two main approaches: traditional machine learning-based methods [27–29] and deep learning-based methods [30–37]. Deep learning-based methods have demonstrated significant advantages over traditional approaches, achieving superior performance in a variety of real-world time series anomaly detection tasks [26]. These approaches leverage the representational power of neural networks—including Convolutional Neural Networks (CNNs), Long Short-Term Memory networks (LSTMs), and Transformers—to capture complex temporal dependencies and non-linear patterns intrinsic to time series data [36,37]. In this study, we will compare the proposed method with several recent deep learning-based approaches [33–35] to assess its relative performance.

### 2.2 ECG anomaly detection

ECG signals, particularly the standard 12-lead ECG data, are multivariate time series that provide essential information for cardiac health monitoring. Building on the advancements in time series anomaly detection, recent research has demonstrated that anomaly detection methods, which can be trained exclusively on normal data, have the potential to identify previously unseen anomalies. This is especially crucial in ECG anomaly detection, where the diversity and rarity of cardiac conditions make the acquisition of sufficient abnormal data a significant challenge. By focusing solely on normal data, anomaly detection methods can effectively reduce the risk of overlooking rare cardiac conditions that may not be well-represented in traditional labeled datasets.

Among the various anomaly detection techniques, generative models have gained significant attention in ECG anomaly detection due to their ability to learn the distribution of normal ECG signals and detect deviations from this learned pattern. Generative Adversarial Network (GAN)-based methods, for example, identify anomalies by measuring the discrepancy between the input ECG signals and those generated by the model [10,11]. A notable example is BeatGAN [12], which excels at capturing local beat-level characteristics, making it particularly effective at detecting subtle, localized abnormalities in ECG signals.

However, ECG anomaly detection remains particularly challenging due to substantial inter-individual and intra-sample variability, as well as the complex nature of anomalies, which can manifest as both global rhythm disturbances and localized morphological irregularities [9,12]. To address these challenges, [9] proposed a multi-scale framework that integrates both local and global features, achieving state-of-the-art performance on the PTB-XL anomaly detection and localization benchmark [9,38]. In addition, considering the clinical need for fast computation and efficiency, [25] proposed a model that integrates both time-series and time-frequency representations of ECG signals. Although their model achieves state-of-the-art results on the PTB-XL detection benchmark, it lacks the ability to localize anomalies, which is critical for many clinical applications. Furthermore, current methods rely on heartbeat segmentation or R-peak detection, which add extra complexity to the model and make it highly sensitive to noise and irregularities in the data, thus limiting their applicability in real-world clinical settings. In addition, there is increasing interest in modeling physiological dynamics directly from continuous biosignals or signal fields without relying on explicit intermediate representations or handcrafted landmarks. For instance, a physics-informed neural network was proposed to estimate respiratory system dynamics directly from pressure–velocity signals, avoiding conventional mesh-based numerical solvers and complex explicit modeling steps [39]. This line of work suggests that robust physiological modeling can be achieved through appropriate model design and learning paradigms rather than explicit feature engineering. This perspective aligns with our approach, as we also avoid heartbeat segmentation or R-peak detection, and instead learn representations directly from raw ECG signals.

### 2.3 Masked autoencoders

Recent advances in deep learning have shifted the focus from increasingly complex model architectures to addressing challenges related to data scarcity [40]. Masked Autoencoders (MAE) [15] have emerged as a powerful self-supervised representation learning framework, showing remarkable success across various visual tasks. This success has prompted efforts to adapt MAE for ECG classification [16–19,22,24]. Notably, [22] proposed an MAE-based multi-label ECG classification method, demonstrating significant performance improvements over previous approaches. However, despite showing promise in capturing certain morphological patterns within ECG signals, MAE’s ability to effectively model both local and global multi-scale features for anomaly detection remains limited [20]. To address this gap, we propose a novel multi-scale MAE-based framework tailored specifically for ECG signals.

## 3 Methodology

**Fig 1:**
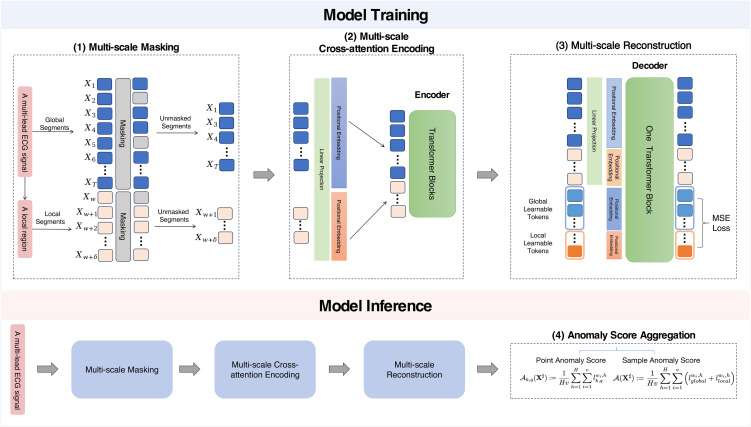
Overview of the proposed framework. (1) Multi-scale Masking: Segments in the global and local regions are masked separately. (2) Multi-scale Cross-attention Encoding: Unmasked segments from both regions are concatenated and fed into a lightweight Transformer-based encoder for cross-attention. (3) Multi-scale Reconstruction: Masked segments in global and local regions are reconstructed using a single-layer Transformer block based on mean square loss after per-segment normalization. (4) Anomaly Score Aggregation: An aggregation strategy enhances sample-level and point-level anomaly scores for anomaly detection and localization, respectively. The Transformer Block denotes the standard Transformer block.

Our proposed framework consists of four key components: (1) multi-scale masking, (2) multi-scale cross-attention encoding, (3) multi-scale reconstruction, and (4) anomaly score aggregation. An overview of the framework is illustrated in Fig 1. In the following, we provide a detailed explanation of each component.

### 3.1 Multi-scale masking

Let a multi-lead ECG signal be denoted as ${𝐗}^{♯}=(X_{k,q}^{♯})\in {\mathbb{R}}^{K\times Q}$, where K represents the number of leads, and Q is the length of the ECG signal. Following [22], we partition the ECG signal ${𝐗}^{♯}$ along the time dimension into a sequence of non-overlapping segments as follows:


$$𝒰:=\{{𝐗}_{1},\cdots ,{𝐗}_{T}\},$$

where T is the total number of segments. Each segment ${𝐗}_{t}=(X_{k,q}^{t})\in {\mathbb{R}}^{K\times (Q/T)}$ represents a subset of the original signal, with $X_{k,q}^{t}=X_{k,\{(t-1cdotQ/T+q\}}^{♯}$ for t=1,…,T, k=1,…,K, and q=1,…,Q/T.

Next, we select multiple consecutive segments from 𝒰 to construct a sequence of local regions ${𝒱}^{w_{1}},\cdots ,{𝒱}^{w_{\nu }}$, where each local region is defined as:


$${𝒱}^{w}:=\{{𝐗}_{w+1},\cdots ,{𝐗}_{w+\delta }\},$$

for $w=w_{1},\ldots ,w_{\nu }$ and $0\leq w_{1}<w_{2}<\ldots <w_{\nu }\leq T-\delta$, where δ is the predefined length of the local region.

During training, for each batch, we randomly select $w\in \{w_{1},\ldots ,w_{\nu }\}$ and separately apply masking to the elements in 𝒰 and ${𝒱}^{w}$. Specifically, given a masking ratio θ, we uniformly sample


$$S^{\prime }:=\min\{\max\{[T\theta ],1\},T-1\}$$ (1)

segments from 𝒰, and


$$R^{\prime }:=\min\{\max\{[\delta \theta ],1\},\delta -1\}$$ (2)

segments from ${𝒱}^{w}$, which are then masked. For notational simplicity, we denote the masked segments as:


$${𝒰}_{mask}=\{{𝐗}_{j_{1}},\cdots ,{𝐗}_{j_{S^{\prime }}}\}$$

and


$${𝒱}_{mask}^{w}=\{{𝐗}_{j_{w,1}},\cdots ,{𝐗}_{j_{w,R^{\prime }}}\},$$

where $j_{s},s=1,\ldots ,S^{\prime }$ and $j_{w,r},r=1,\ldots ,R^{\prime }$ are randomly chosen from the index sets {1,…,T} and {w+1,…,w+δ}, respectively.

Similarly, the unmasked segments are denoted as:


$${𝒰}_{unmask}=\{{𝐗}_{i_{1}},\cdots ,{𝐗}_{i_{S}}\}$$

and


$${𝒱}_{unmask}^{w}=\{{𝐗}_{i_{w,1}},\cdots ,{𝐗}_{i_{w,R}}\},$$

where $i_{s},s=1,\ldots ,S$ and $i_{w,r},r=1,\ldots ,R$ represent the indices of the unmasked segments. Using these notations, we can express the total set of segments as:


$$𝒰={𝒰}_{unmask}\cup {𝒰}_{mask},\;\text{with}\;{𝒰}_{unmask}\cap {𝒰}_{mask}=\emptyset ,$$

and


$${𝒱}^{w}={𝒱}_{unmask}^{w}\cup {𝒱}_{mask}^{w},\;\text{with}\;{𝒱}_{unmask}^{w}\cap {𝒱}_{mask}^{w}=\emptyset .$$

Here, ${𝒰}_{unmask}$ and ${𝒱}_{unmask}^{w}$ are fed into the encoder to achieve multi-scale cross-attention, while ${𝒰}_{mask}$ and ${𝒱}_{mask}^{w}$ serve as the reconstruction targets.

### 3.2 Multi-scale cross-attention encoding

We introduce a self-attention mechanism to model the relationships between global and local features. To achieve this, we first concatenate the unmasked elements from ${𝒰}_{unmask}$ and ${𝒱}_{unmask}$. To preserve sequence order information, we adopt the approach in [22], using learnable positional embeddings. However, applying standard positional embeddings without distinguishing between local and global features could lead to the model overlooking their positional differences. To address this, we introduce distinct positional embeddings for local and global features, enabling the model to better capture and differentiate the unique characteristics of each feature set.

We now describe the encoding module in detail. Denote the layer normalization [41], multi-headed self-attention, and multi-layer perceptron (MLP) blocks, as introduced in [42], by LN(·), MSA(·), and MLP(·), respectively. For simplicity, let ${𝐱}_{i_{s}}^{⊤}$ and ${𝐱}_{i_{w,r}}^{⊤}\in {\mathbb{R}}^{KQ/T}$ represent the vectorized forms of ${𝐗}_{i_{s}}$ for s=1,…,S and ${𝐗}_{i_{w,r}}$ for r=1,…,R. Let D denote the latent vector size. Define the linear projection matrix $𝐄\in {\mathbb{R}}^{(KQ/T)\times D}$, the auxiliary token ${𝐱}_{aux}^{⊤}\in {\mathbb{R}}^{D}$, and the learnable positional embedding vector ${𝐞}_{pos}=({𝐞}_{0},{𝐞}_{1},\cdots ,{𝐞}_{T},{𝐞}_{T+1},\ldots ,{𝐞}_{T+\delta }{)}^{⊤}\in {\mathbb{R}}^{D(T+\delta +1)}$. Here, ${𝐞}_{t}^{⊤}\in {\mathbb{R}}^{D},t=0,1,\ldots ,T$ are used to preserve the sequential order information for global features, while ${𝐞}_{T+t}^{⊤}\in {\mathbb{R}}^{D},t=1,\ldots ,\delta$ are employed to encode local features.

Only the unmasked segments from 𝒰 and ${𝒱}^{w}$ are passed through the model. The input representation is defined as:


$$\begin{array}{c} \begin{array}{cc} {𝐳}_{0}= & \left[{𝐱}_{aux};{𝐱}_{i_{1}}𝐄;\cdots ;{𝐱}_{i_{S}}𝐄;{𝐱}_{i_{w,1}}𝐄;\cdots ;{𝐱}_{i_{w,R}}𝐄\right]\\ & +[{𝐞}_{0};{𝐞}_{i_{1}};\cdots ;{𝐞}_{i_{S}};{𝐞}_{T+i_{w,1}};\cdots ;{𝐞}_{T+i_{w,R}}], \end{array}\; \end{array}$$

where ${𝐱}_{i_{s}}𝐄$ and ${𝐞}_{i_{s}}$ denote the projections of the unmasked global segments and their corresponding positional embeddings, and ${𝐱}_{i_{w,r}}𝐄$ and ${𝐞}_{T+i_{w,r}}$ represent the projections of the unmasked local segments along with their respective embeddings.

The encoding process consists of multiple layers of self-attention and MLP blocks:


$${𝐳}_{l}^{\prime }=MSA(LN({𝐳}_{l-1}))+{𝐳}_{l-1},\;l=1,\ldots ,L,$$

$${𝐳}_{l}=MLP(LN({𝐳}_{l}^{\prime }))+{𝐳}_{l}^{\prime },\;l=1,\ldots ,L,$$

where L denotes the number of transformer blocks.

Finally, the output of the encoder is given by:


$${𝐳}_{L}=[{𝐳}_{L}^{0};{𝐳}_{i_{1}}^{L};\cdots ;{𝐳}_{i_{S}}^{L};{𝐳}_{i_{w,1}}^{L};\cdots ;{𝐳}_{i_{w,R}}^{L}],$$

where ${𝐳}_{L}^{0}\in {\mathbb{R}}^{D}$ represents the encoded auxiliary token, ${𝐳}_{i_{s}}^{L}\in {\mathbb{R}}^{D}$ are the encoded unmasked global segments, and ${𝐳}_{i_{w,r}}^{L}\in {\mathbb{R}}^{D}$ are the encoded unmasked local segments. These encoded representations are subsequently used to reconstruct the global and local features, respectively.

### 3.3 Multi-scale reconstruction

In this section, we present the multi-scale reconstruction strategy that employs a Transformer-based decoder. This decoder helps encourage the encoder to learn meaningful wave shape features. Specifically, we adopt a one-layer Transformer decoder. Let $D^{\prime }$ denote the latent vector size. We define the learnable components as follows: ${𝐄}^{\prime }\in {\mathbb{R}}^{D\times D^{\prime }}$, ${𝐄}_{0}\in {\mathbb{R}}^{D^{\prime }\times (KQ/T)}$, ${𝐞}_{m}^{⊤}\in {\mathbb{R}}^{D^{\prime }}$, and the positional embeddings ${𝐞}_{pos}^{\prime }=({𝐞}_{1}^{\prime },\cdots ,{𝐞}_{T}^{\prime },{𝐞}_{T+1}^{\prime },\cdots ,{𝐞}_{T+\delta }^{\prime }{)}^{⊤}\in {\mathbb{R}}^{(T+\delta )D^{\prime }}$. Here, ${𝐞}_{t}^{\prime }\in {\mathbb{R}}^{D^{\prime }}$ for t=1,…,T corresponds to the positional embeddings for global features, and ${𝐞}_{T+t}^{\prime }\in {\mathbb{R}}^{D^{\prime }}$ for t=1,…,δ serves as the positional embeddings for local features. Additionally, ${𝐞}_{m}^{⊤}$ represents the embeddings for the masked segments.

The decoder can be formulated as follows:


$$\begin{array}{c} \begin{array}{cc} {\tilde{𝐳}}_{0} & =[{𝐳}_{L}^{i_{1}}{𝐄}^{\prime };\cdots ;{𝐳}_{L}^{i_{S}}{𝐄}^{\prime };{𝐳}_{L}^{i_{w,1}}{𝐄}^{\prime };\cdots ;{𝐳}_{L}^{i_{w,R}}{𝐄}^{\prime };\\ & \;{𝐞}_{m};\cdots ;{𝐞}_{m}]+[{𝐞}_{i_{1}}^{\prime };\cdots ;{𝐞}_{i_{S}}^{\prime };{𝐞}_{i_{w,1}}^{\prime };\cdots ;{𝐞}_{i_{w,R}}^{\prime };\\ & \;{𝐞}_{j_{1}}^{\prime };\cdots ;{𝐞}_{j_{S^{\prime }}}^{\prime };{𝐞}_{j_{w,1}}^{\prime };\cdots ;{𝐞}_{j_{w,S^{\prime }}}^{\prime }], \end{array}\; \end{array}$$

$${\tilde{𝐳}}_{1}^{\prime }=MSA(LN({\tilde{𝐳}}_{0}))+{\tilde{𝐳}}_{0},$$

$${\tilde{𝐳}}_{1}=MLP(LN({\tilde{𝐳}}_{1}^{\prime }))+{\tilde{𝐳}}_{1}^{\prime }.$$

Here, ${\tilde{𝐳}}_{1}^{\prime }$ is given by


$${\tilde{z}}_{1}^{'}=[{\tilde{z}}_{1}^{i_{1}};\cdots ;{\tilde{z}}_{1}^{i_{S}};{\tilde{z}}_{1}^{i_{w,1}};\cdots ;{\tilde{z}}_{1}^{i_{w,R}};\;\;{\tilde{z}}_{1}^{j_{1}};\cdots ;{\tilde{z}}_{1}^{j_{S^{'}}};{\tilde{z}}_{1}^{j_{w,1}};\cdots ;{\tilde{z}}_{1}^{j_{w,R^{'}}}],$$

where each $({\tilde{𝐳}}_{1}^{i_{s}}{)}^{⊤},({\tilde{𝐳}}_{1}^{j_{s^{\prime }}}{)}^{⊤},({\tilde{𝐳}}_{1}^{i_{w,r}}{)}^{⊤},({\tilde{𝐳}}_{1}^{j_{w,r^{\prime }}}{)}^{⊤}\in {\mathbb{R}}^{D^{\prime }}$ for s=1,…,S, $s^{\prime }=1,\ldots ,S^{\prime }$, r=1,…,R, and $r^{\prime }=1,\ldots ,R^{\prime }$. The segments ${\tilde{𝐳}}_{1}^{j_{s^{\prime }}}$ and ${\tilde{𝐳}}_{1}^{j_{w,r^{\prime }}}$ are used to reconstruct the global and local masked segments, respectively.

The decoder outputs are obtained by:


$${\tilde{𝐱}}_{j_{s^{\prime }}}={\tilde{𝐳}}_{1}^{j_{s^{\prime }}}{𝐄}_{0},\;s^{\prime }=1,\ldots ,S^{\prime },$$

and


$${\tilde{𝐱}}_{j_{w,r^{\prime }}}={\tilde{𝐳}}_{1}^{j_{w,r^{\prime }}}{𝐄}_{0},\;r^{\prime }=1,\ldots ,R^{\prime }.$$

During training, the objective is to reconstruct the normalized values of the masked global and local segments. We define the reconstruction loss for the global and local features as:


$$l_{\text{global}}=\sum_{s^{\prime }=1}^{S^{\prime }}{\left\|{\tilde{𝐱}}_{j_{s^{\prime }}}-f({𝐱}_{j_{s^{\prime }}})\right\|}_{2}^{2},$$

and


$$l_{\text{local}}^{w}=\sum_{r^{\prime }=1}^{R^{\prime }}{\left\|{\tilde{𝐱}}_{j_{w,r^{\prime }}}-f({𝐱}_{j_{w,r^{\prime }}})\right\|}_{2}^{2},$$

where ${𝐱}_{j_{s^{\prime }}}$ and ${𝐱}_{j_{w,r^{\prime }}}\in {\mathbb{R}}^{QW/T}$ are the vectorized forms of the global and local segments ${𝐗}_{j_{s^{\prime }}}$ and ${𝐗}_{j_{w,r^{\prime }}}$, and $f:{\mathbb{R}}^{QW/T}\to {\mathbb{R}}^{QW/T}$ is a predefined per-segment normalization function as specified in [22]. The final loss function is then the sum of the global and local reconstruction losses:


$$l^{w}=l_{\text{global}}+l_{\text{local}}^{w}.$$

### 3.4 Anomaly score aggregation

In the anomaly detection framework, each test sample ${𝐗}^{♯}$ undergoes a sequence of forward passes, where the masking segments are determined randomly in each pass. To ensure that segments within the local region are reconstructed with high probability, we evaluate the test sample through H independent forward passes. Here, H is a predefined constant, which ensures that a segment is masked with the probability:


$$1-{\left(\frac{R}{\delta }\right)}^{H},$$ (3)

where R represents the number of masked segments and δ is the total number of segments.

To further improve reconstruction accuracy, we leverage multi-scale cross-attention to cover all local regions, including ${𝒱}^{w_{1}},\cdots ,{𝒱}^{w_{v}}$. For each local region ${𝒱}^{w_{i}}$ and each forward pass h, we denote the corresponding reconstruction loss as $l_{local}^{w_{i},h}$, for i=1,…,v and h=1,…,H. Additionally, since the global features may also vary across different passes and regions, we use $l_{global}^{w_{i},h}$ to denote the loss associated with the global features for the same i and h.

The anomaly score for the test sample ${𝐗}^{♯}$ is then defined as the average of the losses across all local regions and forward passes:


$$𝒜({𝐗}^{♯}):=\frac{1}{Hv}\sum_{h=1}^{H}\sum_{i=1}^{v}\left(l_{global}^{w_{i},h}+l_{local}^{w_{i},h}\right).$$ (4)

For localization of anomalies, the anomaly score for a specific signal point, denoted as $X_{k,q}^{♯}$, corresponds to the part of the anomaly score in (4) that is related to that signal point. Specifically, the global and local loss terms $l_{global}^{w_{i},h}$ and $l_{local}^{w_{i},h}$ are aggregated over a subset of signal points. By summing the contributions related to $X_{k,q}^{♯}$, we define the localized anomaly score $l_{k,q}^{w_{i},h}$ for each forward pass h and local region $w_{i}$. The final anomaly score for the signal point $X_{k,q}^{♯}$ is given by:


$${𝒜}_{k,q}({𝐗}^{♯}):=\frac{1}{Hv}\sum_{h=1}^{H}\sum_{i=1}^{v}l_{k,q}^{w_{i},h}.$$ (5)

## 4 Experiments

This section presents an evaluation of the proposed method using the PTB-XL anomaly detection and localization benchmark [9], which offers a comprehensive tool for ECG-based anomaly detection tasks. We first introduce the dataset and then the experiment setting and results. Additional results, including the relationship between anomaly scores and ECG diagnoses, comparisons with a traditional machine learning method, and the cross-dataset evaluation, are provided in S1 Appendix.

### 4.1 Dataset

The PTB-XL anomaly detection and localization benchmark is built by [9], based on the original PTB-XL dataset [38]. The original dataset is a widely used open-source dataset for evaluating ECG model performance, notable for its relatively large sample size and high-quality annotations. It comprises 21,837 clinical 12-lead ECG records of 10 seconds length and 500 Hz sampling rate, each recording have patient-level annotations with 71 distinct ECG statements. For the anomaly detection benchmark, we follow the anomaly detection and localization benchmark protocols proposed in [9]. The anomaly detection training set was constructed as a subset of the PTB-XL training set [38], consisting of 8,167 ECG recordings labeled as normal, with all abnormal recordings excluded. The detection test set was derived from the PTB-XL test set and includes 912 normal and 1,248 abnormal recordings. Recordings labeled as “NORM” are regarded as normal, whereas all remaining recordings with at least one diagnostic label are treated as abnormal, covering a wide range of cardiovascular abnormalities [38]. The localization test set comprises 400 abnormal recordings from the PTB-XL test set, with point-level signal annotations across 22 abnormality types provided by two experienced cardiologists. The model was trained on the anomaly training set and evaluated on both the detection and localization test sets. The PTB-XL detection and localization benchmark, including train-test splits and annotation files, is publicly available at https://github.com/MediaBrain-SJTU/ECGAD.

### 4.2 Implementation details

In our experiments, we selected the segment size by considering the typical durations of major ECG waveforms, as the P wave, T wave, and QRS complex usually last between 0.05 and 0.25 seconds [43,44]. Since anomaly detection in our framework relies on the reconstruction error of segments, we set the segment length to 125 samples, which corresponds to 0.25 seconds at a 500 Hz sampling rate, ensuring that each segment preserves the major morphological information of these waveforms. Given that each ECG signal is sampled for 10 seconds (i.e., 5000 time steps), applying the non-overlapping splitting strategy results in a sequence length of T=40. We set δ=4 and define the local regions at the points 1, 5, 9, 13, 17, 21, 25, 29, 33, excluding the segments at the beginning and end of the sequence, similar to [9]. The masking ratio is set to θ=25%, and the encoder consists of L=3 layers with 16 self-attention heads and a latent dimension of D=64. The decoder has the same latent dimension of $D^{\prime }=64$ with 2 self-attention heads. Training uses the AdamW optimizer with a cosine annealing learning rate schedule and a batch size of 256, running for 300 epochs with a warm-up of 40 epochs. For inference, we select H=4 to ensure that each segment in the local regions is masked with at least 99% probability. Performance is evaluated using the Area Under the Receiver Operating Characteristic Curve (AUC), following [9] and [25].

The experiments are conducted on a server equipped with an NVIDIA Tesla V100 GPU and an Intel Xeon Gold 6130 CPU. Following [16], we report the total training time required to complete the optimization of the proposed model, which provides insights into the cost of model development. Considering that model deployment in healthcare institutions may vary, especially in resource-limited settings, we further quantify the computational complexity per sample using GFLOPs (giga floating-point operations) during inference. This metric measures the number of arithmetic operations required by the model to process a single input and can serve as a reference for potential clinical deployment.

### 4.3 Comparisons with state-of-the-arts

We compare our proposed method with several state-of-the-art time-series anomaly detection approaches, including TranAD [33], AnoTran [34], TSL [35], BeatGAN [12], MCF [9] and TSRNet [25]. The results of TranAD, AnoTran, TSL and MCF are excerpted from [9], while that of TSRNet is excerpted from [25]. As shown in Table 1, both MCF and our method significantly outperform baseline models in anomaly detection and localization, with our method achieving comparable detection performance and slightly better localization accuracy. This demonstrates our method’s ability to effectively capture both global and local features of ECG signals, offering improved robustness and precision over existing solutions.

**Table 1:** Comparison of methods.

Method	Detection	Localization
TranAD [33]	0.788	0.685
AnoTran [34]	0.762	0.641
TSL [35]	0.757	0.509
BeatGAN [12]	0.799	0.715
TSRNet [25]	**0.860**	–
MCF [9]	**0.860**	0.747
MMAE-ECG	**0.860**	**0.749**

Table 2 further highlights the computational efficiency of our method. Unlike MCF, which requires R-peak detection during preprocessing, our method eliminates this step, simplifying data preparation. In terms of computational complexity (GFLOPs), MCF requires 45.108 GFLOPs per inference, computed as 1.253×12×3, where 1.253 represents the GFLOPs per forward pass, *12* corresponds to the number of R-peaks, and *3* accounts for feed-forward operations. Specifically, MCF performs approximately 36 forward passes, based on the median number of R-peaks detected by its implementation [9], with each pass requiring 1.253 GFLOPs. In contrast, our method requires only 0.576 GFLOPs per inference, computed as 0.016×9×4, where 0.016 denotes the GFLOPs per forward pass, *9* represents the number of local regions, and *4* corresponds to the number of aggregation operations (*H*). This results in an approximately 78× reduction in computational complexity compared to MCF (0.576 GFLOPs vs. 45.108 GFLOPs), significantly lowering resource demands. Moreover, our approach features a substantially smaller model size (0.398M parameters vs. 7.086M) and a dramatically faster training time (0.225 hours vs. 9.537 hours). The reported training time is the median of five independent runs, conducted on a server equipped with an NVIDIA Tesla V100 GPU and an Intel Xeon Gold 6130 CPU. These improvements in computational efficiency and model complexity make our method particularly well-suited for deployment in resource-constrained environments, enhancing its practical applicability in real-world clinical settings.

**Table 2:** Comparison of computational complexity and model requirements.

Metric	MCF	MMAE-ECG
R-peak Detection	Required	**Not required**
Inference Complexity (GFLOPs)	≈ 45.108	**0.576**
Parameters (M)	7.086	**0.398**
Model Type	Convolutional-based	Transformer-based
Training Time (h)	9.537	**0.225**

### 4.4 Additional performance metrics

**Fig 2:**
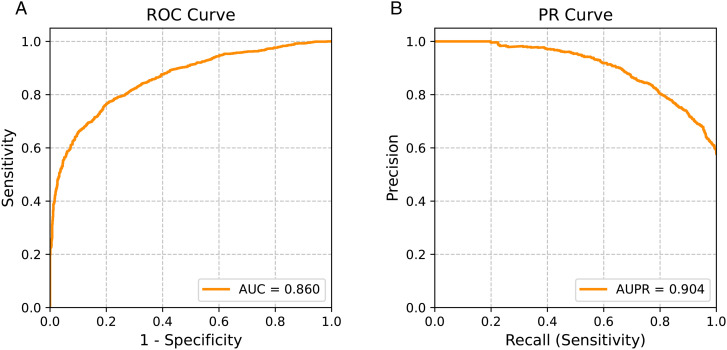
(A) Receiver operating characteristic (ROC) curve and (B) precision–recall (PR) curve of the proposed method, illustrating its discrimination ability across varying thresholds.

To provide a more comprehensive evaluation, we further report precision, recall (sensitivity), F1-score, and specificity in addition to AUC. Fig 2 illustrates the Receiver Operating Characteristic (ROC) and Precision–Recall (PR) curves of the proposed anomaly detection method, offering an overall view of its discrimination ability across varying thresholds.

To complement these results, Table 3 summarizes the detailed performance metrics at different recall (sensitivity) levels ranging from 0.050 to 0.950, thereby covering a wide operating spectrum. For example, when sensitivity is fixed at 0.900, the corresponding precision is 0.729 and the F1-score reaches 0.806. These findings highlight that the method maintains a relatively favorable balance between sensitivity and precision under different decision thresholds, which may be of practical value for real-world deployment where clinical requirements often vary.

**Table 3:** Detailed performance metrics of the proposed method at different recall (sensitivity) levels, including corresponding precision, F1-score, and specificity.

Recall	Precision	F1-score	Specificity
0.050	1.000	0.095	1.000
0.100	1.000	0.182	1.000
0.200	0.996	0.332	0.999
0.300	0.982	0.459	0.992
0.400	0.971	0.566	0.984
0.500	0.954	0.656	0.967
0.600	0.919	0.726	0.928
0.700	0.865	0.774	0.851
0.800	0.804	0.802	0.734
0.900	0.729	0.806	0.543
0.950	0.680	0.792	0.388

### 4.5 Visualization for anomaly localization

**Fig 3:**
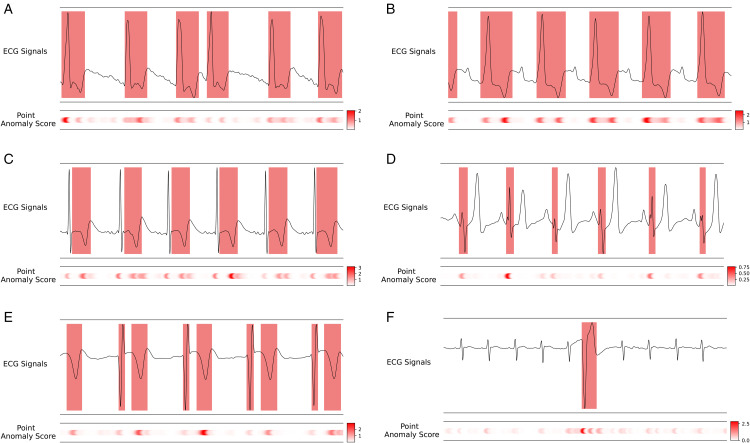
Examples of anomaly localization on the PTB-XL dataset across different types of ECG abnormalities. Ground truth regions, annotated by cardiologists, are highlighted with red boxes on the ECG signals, while the corresponding anomaly localization results based on the point-level anomaly score (defined in 5) of the proposed method are shown below. Detailed descriptions are provided in S1 Appendix.

To further demonstrate the effectiveness of MMAE-ECG in anomaly localization, we present visualization results on representative samples from the PTB-XL benchmark, as shown in Fig 3. These examples cover a diverse range of ECG abnormalities, as annotated by experienced cardiologists [9], with detailed descriptions provided in S1 Appendix. As illustrated in Fig 3, the proposed method effectively highlights abnormal regions across different ECG leads. These visualizations provide an intuitive interpretation of the model‘s predictions and serve as a form of attribution explanation [45], indicating which input features contributed most to the detected anomalies and why the model made such decisions. Together, these results suggest that MMAE-ECG could assist clinicians in rapid and accurate anomaly localization in real-world clinical scenarios.

### 4.6 Ablation study

We conduct ablation studies to systematically evaluate the contribution of each design choice in our model, using the PTB-XL anomaly detection benchmark, which includes patients with diverse characteristics. Specifically, we investigate the following key aspects:

- The impact of multi-scale region utilization.
- The effectiveness of the local positional embedding.
- The influence of the multi-scale masking strategy.
- The necessity of the masked segment-based loss function.
- The effect of varying masking ratios.
- The influence of different aggregation strategies during inference.

We design a series of experiments to evaluate these aspects. The results for experiments **a** to **d** are summarized in Table 4. Specifically:

**Table 4:** Ablation study results for different model configurations.

Configuration	AUC
MMAE-ECG	**0.860**
**(a)** Global region only	0.825
**(a)** Local region only	0.793
**(b)** Global positional embedding applied to local region	0.847
**(c)** Single mask (vs. Multi-scale mask)	0.845
**(d)** Loss computed on all segments	0.731

- For **a**, we evaluate the model‘s performance by removing either the local region or the global region in our framework.
- For **b**, we replace our specially-designed local positional embedding with the corresponding positional embedding used for global region 𝒰.
- For **c**, we replace the multi-scale masking strategy with a single masking approach, where several segments are randomly masked from the concatenated global and local features, potentially leaving all the local segments unmasked.
- For **d**, we modify the loss function to compute the loss over all segments, rather than just the masked segments.

The results of these experiments show a significant degradation in anomaly detection performance when the proposed settings are not applied, as detailed in Table 4.

**Fig 4:**
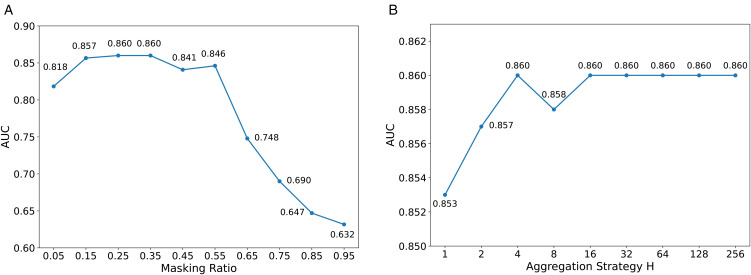
Ablation study results for different masking ratios and values of *H.* In **(A)**, the x-axis is for different masking ratio *θ* used in (1) and (2). While in **(B)**, the x-axis represents *H* used in the probability (3).

Experiments **e** and **f** are shown in Fig 4. Specifically:

- For **e**, we evaluate the algorithm under different masking ratios, ranging from 0.5 to 0.95.
- For **f**, we examine the influence of varying the aggregation strategy *H*, with values including 1, 2, 4, 8, 16, 32, 64, 128, and 256 during inference.

Fig 4A shows that masking ratios between 0.15 and 0.35 yield optimal performance, which is consistent with previous findings in ECG multi-label classification [22]. Fig 4B illustrates that performance exhibits a slight increase as *H* grows and stabilizes when *H* becomes sufficiently large.

## 5 Discussion

This study introduces a novel multi-scale masked autoencoder (MAE) framework for electrocardiogram (ECG) anomaly detection and localization, achieving state-of-the-art performance on the recently released PTB-XL benchmark [9]. By jointly modeling global and local temporal dependencies within an end-to-end Transformer-based architecture, our method departs from traditional ECG anomaly detection pipelines that rely heavily on heartbeat segmentation or R-peak detection [9,25].

One advantage of the proposed approach lies in its simplicity and robustness: the elimination of heartbeat segmentation and R-peak detection streamlines preprocessing, substantially reducing complexity and potential failure points in real-world clinical settings. This property is particularly relevant in high-throughput environments, where more than 300 million ECG recordings are processed annually [46]. During our experiments, we noted that certain samples in PTB-XL [38] were excluded from prior benchmarks [9], potentially due to failures in widely used R-peak detection algorithms implemented in public Python libraries. While the exact cause of these exclusions cannot be definitively established, this observation underscores a broader challenge—existing detection and segmentation methods can be brittle when applied across diverse ECG morphologies. Furthermore, previous studies have shown that R-peak detection and ECG delineation results can vary considerably across different algorithms and devices [47,48], potentially introducing additional variability into downstream models [49]. In contrast, the proposed method deliberately bypasses R-peak detection and heartbeat segmentation, operating directly on raw ECG signals. This design choice reduces dependence on fragile preprocessing pipelines and simplifies the overall workflow. Although this strategy is not intended to guarantee direct performance gains, it is expected to improve robustness across heterogeneous ECG morphologies, noise conditions, and device settings, which is critical for real-world clinical deployment.

From an interpretability perspective, the anomaly localization produced by the proposed framework provides a form of attribution-based explanation. The reconstruction paradigm enables the model to assign anomaly scores at fine temporal resolution, as defined in (5), thereby highlighting localized regions in the ECG signal that contribute most to the detected abnormality. As illustrated in Fig 3, such visualizations offer an intuitive way to inspect model predictions by mapping anomalous responses back to the original signal, serving as a practical form of post-hoc interpretability [45]. Given that many ECG-related tasks focus on identifying abnormal waveform morphologies or transient deviations, localized anomaly visualizations can assist clinicians in rapidly inspecting suspicious regions, potentially improving review efficiency. We emphasize that this interpretability is complementary to traditional waveform-level analysis and is not intended to replace clinical judgment.

In addition to its robustness and the interpretability afforded by anomaly localization, the proposed model is highly efficient. In general, the hyperparameters were determined through a combination of manual tuning and reference to prior literature [9,22]. Since the P, T waves and QRS complex in an ECG signal typically lasts 0.05–0.25 seconds [43,44], we set the segment length to 125 samples (0.25 seconds at a 500 Hz sampling rate) to capture the major morphology of these fundamental waveforms. In a recent study on ECG classification with a private dataset [22], they conducted comprehensive experiments using the following hyperparameters: masking ratio θ=25%, latent dimension D=64, batch size of 256, 300 training epochs, and the AdamW optimizer with a cosine annealing learning rate schedule. Unlike the previous study [22], our task retains the decoder in the downstream task. Therefore, we set the latent dimension of the decoder equal to that of the encoder. Although these settings may not be exactly optimal, they achieve near-optimal performance while substantially reducing computational cost. In addition, the implementation code of a prior work on ECG anomaly detection considered local regions at positions similar to our setting, while excluding boundary segments [9]. Based on our own design, we set the forward pass to H=4, which ensures that each segment in the local regions is masked with at least 99% probability. With only 0.398 million parameters and 0.576 GFLOPs per inference pass, it requires approximately 1/78 of the FLOPs and 1/18 of the trainable parameters compared to the previous state-of-the-art ECG detection and localization approach (7.086 million parameters and 45.108 GFLOPs) [9]. This lightweight design facilitates deployment in resource-constrained settings and accelerates both training and inference, further enhancing clinical applicability.

A distinctive methodological contribution of this work is the integration of local feature modeling through the concatenation of signal subsegments with positional embeddings, combined with a multi-scale masking strategy. To our knowledge, this is the first application of such techniques within the MAE framework for ECG analysis. Despite its conceptual simplicity, this approach has demonstrated notable empirical benefits. Ablation studies confirm that each component—including the multi-scale masking—contributes meaningfully to performance. Our experiments reveal that multi-scale strategies substantially improve the model’s capacity to extract informative representations across temporal resolutions. While prior studies have argued that MAE architectures may be suboptimal for anomaly detection tasks [20], our findings suggest that incorporating multi-scale mechanisms and local feature attention can overcome these limitations and lead to significant performance gains.

Beyond anomaly detection and localization, our approach has broader potential in ECG analysis. Recent work has demonstrated that Transformer-based architectures relying primarily on global features can achieve competitive performance in multi-label classification in 2023 [22]. Prior to its official publication, Fuwai Hospital had already built upon this model to develop an AI-ECG system that significantly enhances diagnostic efficiency in clinical practice and has been operating stably to date (see the WeChat Official Account of Fuwai Hospital at this link). An open question for future research is whether integrating local feature modeling, as proposed here, could further enhance classification performance. Investigating this direction may yield more comprehensive models that jointly exploit global and local information, ultimately improving the accuracy and reliability of automated ECG interpretation. Given the potential for similar paradigms in other physiological data, extending this framework to signals such as photoplethysmography (PPG) also represents a promising avenue for future research.

Although the method has been validated on PTB-XL, one of the most commonly used benchmark datasets covering a wide range of ECG abnormalities, external validation has not yet been conducted. Consequently, the potential influence of factors such as differences in racial populations and recording protocols remains unclear. Future work should evaluate the framework on ECG datasets from multiple centers to better assess its robustness and generalizability. While our study focuses on the 12-lead ECG, which is the clinical gold standard and provides the most comprehensive information, it is worth noting that reduced-lead recordings from wearable or mobile devices also represent an important application scenario. In such cases, differences in signal quality and reduced spatial information may pose additional challenges, requiring further adaptation of the framework. Furthermore, although PTB-XL is relatively large, some rare ECG abnormalities are not represented in this dataset. Larger and more comprehensive datasets would further support the evaluation and demonstrate the potential of ECG anomaly detection methods. From a computational perspective, the proposed framework is relatively lightweight, requiring substantially fewer parameters and FLOPs than prior leading approaches [9], and its efficiency can be further improved by tuning hyper-parameter *H*. For instance, reducing *H* from 4 to 1 decreases FLOPs for inference to one quarter, at the cost of only a 0.8% drop in AUC. Exploring other efficiency-oriented modifications, such as reducing the latent dimension as in [22], may offer additional gains, though these directions warrant further empirical validation.

Finally, privacy and security remain critical considerations in the handling of sensitive ECG data. Although the present study primarily focuses on algorithmic development, both training and inference stages should carefully address these concerns. For instance, when training data are collected from multiple centers, safeguards such as data anonymization, secure communication protocols, and privacy-preserving learning paradigms (e.g., federated learning [50]) should be adopted. During inference, edge deployment offers a practical solution to minimize data transmission. If remote servers are employed, additional security mechanisms such as encryption should be incorporated to protect patient information.

## 6 Conclusion

This paper presents a lightweight and robust multi-scale masked autoencoder framework for ECG anomaly detection and localization. By eliminating the need for R-peak detection and heartbeat segmentation, the proposed approach simplifies preprocessing and enhances robustness in clinical applications. Through the integration of multi-scale masking and attention mechanisms, the model effectively captures both global and local temporal dependencies, achieving superior performance on the PTB-XL benchmark. Notably, it reduces computational complexity by approximately 1/78 in FLOPs and 1/18 in trainable parameters compared to the previous leading method, supporting its suitability for deployment in resource-constrained environments. Potential directions for future work include: (i) extending this framework into a general pre-training strategy by leveraging the multi-scale design to initialize the encoder and subsequently fine-tuning it on various ECG classification and regression tasks; and (ii) evaluating the method on more diverse datasets, including multi-center 12-lead ECGs, reduced-lead ECGs, and other physiological signals such as photoplethysmography (PPG).

## Supporting information

S1 Appendix Supplementary methods and additional analyses. (PDF)
