# Supplementary material for: A lightweight and robust method for electrocardiogram anomaly detection and localization using multi-scale masked autoencoder
Source: PLoS One. 2026 Mar 17;21(3):e0343571. doi: 10.1371/journal.pone.0343571 (PMC12995306; doi:10.1371/journal.pone.0343571)
Supplement: S1 Appendix — (PDF) [file pone.0343571.s001.pdf]

# A lightweight and robust method for electrocardiogram anomaly detection and localization using multi-scale masked autoencoder

Ya Zhou[1,*], Yujie Yang[1], Jianhuang Gan[1], Xiangjie Li[2], Jing Yuan[1], Wei Zhao[3*]

**1** Department of Information Center, Fuwai Hospital, Chinese Academy of Medical Sciences and Peking Union Medical College, Beijing, 100037, China
**2** National Clinical Research Center for Cardiovascular Diseases, Fuwai Hospital, Chinese Academy of Medical Sciences and Peking Union Medical College, National Center for Cardiovascular Diseases, Beijing, 100037, China
**3** Center for Health Statistics and Information, National Health Commission of the People's Republic of China, Beijing, 100044, China.

These authors contributed equally to this work.
* zhaowei@nhc.gov.cn, zhouya@fuwai.com

# A Appendix

## A.1 Details of Visualization Examples

Figure 3 illustrates a portion of ECG signals from different leads of examples in the PTB-XL localization benchmark [1]. In particular, it shows the AVR lead from the 212rd sample (A), the V1 lead from the 225th sample (B), the V4 lead from the 230th sample (C), the V1 lead from the 234th sample (D), the V2 lead from the 376th sample (E), and the V2 lead from the 389th sample (F). By mapping these samples to the SCP-ECG statements in the PTB-XL dataset [2], we identify the corresponding clinical annotations: the 212rd, 225th, 230th, 234th, 376th and 389th samples are labeled with [complete left bundle branch block, first degree AV block, premature ventricular contractions], [complete right bundle branch block, left posterior fascicular block, first degree AV block, right ventricular hypertrophy], [left ventricular hypertrophy, non-specific ischemic, incomplete right bundle branch block, first degree AV block], [inferolateral myocardial infarction, anteroseptal myocardial infarction], [anteroseptal myocardial infarction, left ventricular hypertrophy, non-specific ischemic, non-specific intraventricular conduction disturbance (block), left atrial overload/enlargement], and [ischemic in anterolateral leads, ischemic in inferior leads, premature ventricular contractions], respectively.

## A.2 Relationship between Anomaly Scores and ECG Diagnoses

Our work focuses on ECG anomaly detection, which differs from the ECG multi-label classification task. Nevertheless, the abnormal cases identified by our model correspond to several clinical annotations in the PTB-XL dataset [2]. In other words, when the model detects an anomaly, the corresponding ECG record typically contains at least one abnormal clinical annotation in PTB-XL. For instance, we used subclass labels [2], and a summary of these clinical annotations, excluding the normal class, is provided in Table A1. Therefore, when our model correctly identifies an abnormal ECG, it is associated with at least one annotation listed in the table.

**Table A1.** ECG Abnormality Subclasses

| Category | Subclasses |
|---|---|
| ECG Abnormalities | AMI, IMI, LMI, PMI, STTC, NST_, ISC_, ISCA, ISCI, _AVB, CLBBB, ILBBB, CRBBB, IRBBB, IVCD, LAFB/LPFB, LVH, RVH, SEHYP, LAO/LAE, RAO/RAE, WPW |

Furthermore, we investigated the relationship between the anomaly scores and the clinical annotations in the PTB-XL dataset. Specifically, we computed the one-vs-all AUC and AUPR for each label. Taking CRBBB as an example, we defined a binary label where the value is 1 if the ECG record is annotated as CRBBB and 0 otherwise. We then evaluated the relationship between this new label and the anomaly scores generated by our model. Interestingly, we found that the anomaly scores achieve high accuracy in identifying multiple disease labels. As shown in Figure A1, the AUC values of several labels exceed 0.8. To further illustrate this finding, Figure 2 presents the ROC and PR curves for CRBBB as an example.

These analyses suggest that although our model is trained solely on normal ECGs, it still exhibits the ability to identify specific cardiovascular abnormalities. This may be because different disease patterns produce distinct waveform deviations, leading to different reconstruction errors.

## A.3 Additional Experiment Using a Traditional Machine Learning Method

We conducted an additional experiment using an SVM-based method [3], which relies on heart rate variability (HRV) features. Specifically, we first performed ECG denoising and detected R-peaks using the computationally efficient and widely adopted peak detection algorithm from NeuroKit2 [4], which integrates multiple validated signal processing techniques. Subsequently, we extracted both time-domain and frequency-domain HRV features to train an SVM classifier.

The SVM-based model achieved an AUC of 0.677, which is significantly lower than that of the proposed method (AUC = 0.860). Although traditional machine learning methods are computationally efficient, their performance remains substantially lower than that of the deep learning approaches presented in Table 1 of the main manuscript, which may reflect the inherent differences in capabilities between these two types of methods.

## A.4 External Dataset Evaluation

To further assess robustness, we conducted an external evaluation using the Chapman–Shaoxing–Ningbo dataset [5], which differs from PTB-XL in acquisition systems and population characteristics.

### A.4.1 External Dataset Construction and Anomaly Definition

The external test set is a subset of the Chapman–Shaoxing–Ningbo dataset [5]. Recordings containing missing values were excluded, and only adult subjects (age $\geq$ 18 years) were included. SNOMED-CT diagnostic labels were mapped to the same diagnostic hierarchy as PTB-XL to ensure diagnostic-level consistency. Following the benchmark protocol proposed in [1], recordings associated with at least one disease-related diagnostic label were treated as abnormal. Recordings annotated exclusively with sinus rhythm (SR; SNOMED-CT code 426783006) were considered

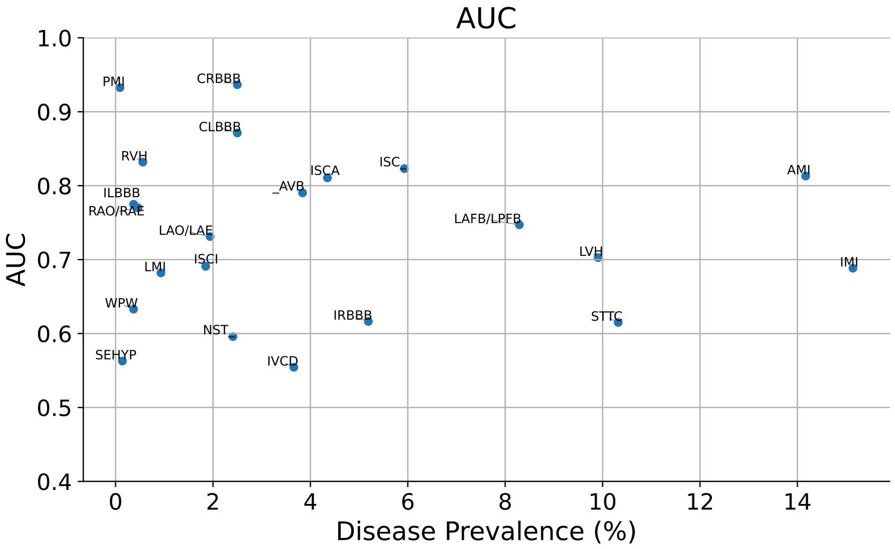


(A)

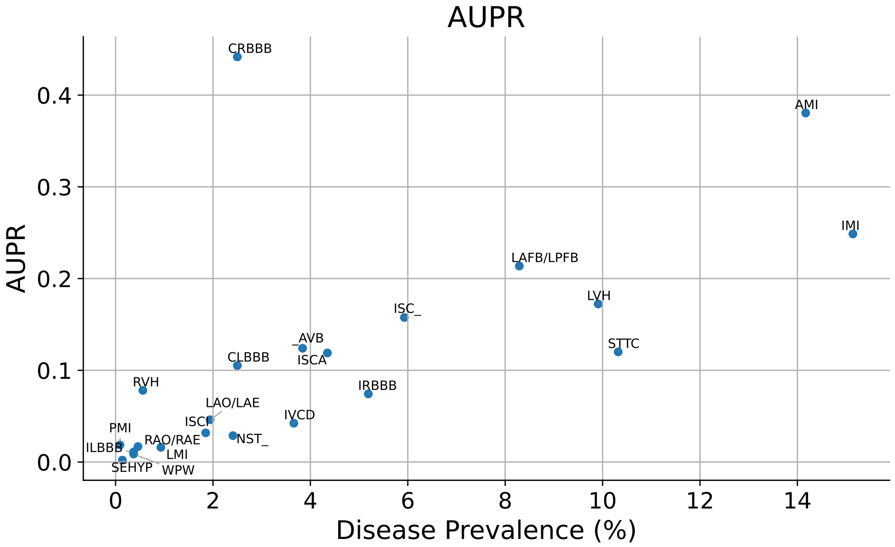


(B)

**Fig A1.** Performance of the proposed model in relation to the prevalence of each subclass label, excluding the normal class. (A) Relationship between area under the receiver operating characteristic curve (AUC) and label prevalence. (B) Relationship between area under the precision–recall curve (AUPR) and label prevalence.

normal, providing a clinically consistent approximation of normality for the external dataset. The resulting test set consists of 17,195 12-lead ECG recordings (5,641 normal, 11,554 abnormal) from patients aged 18–89 years, with 10-second recordings.

### A.4.2 Results on the External Dataset

Without any retraining or fine-tuning, the proposed model trained on the PTB-XL-based anomaly training set was directly evaluated on the external dataset, achieving an AUC of 0.814 and an AUPR of 0.904. It is worth mentioning that the

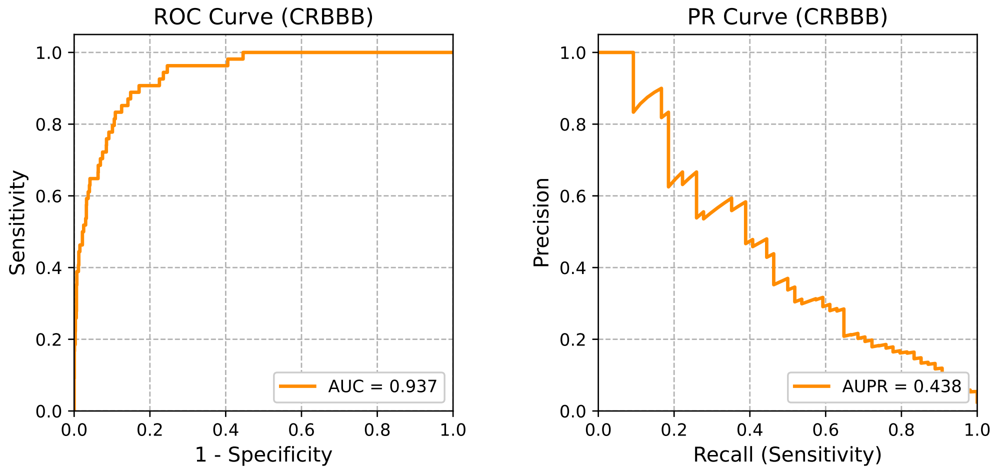


(A) (B)

**Fig A2.** (A) Receiver operating characteristic (ROC) curve and (B) precision–recall (PR) curve of the proposed method for the CRBBB label, taken as an example to illustrate the relationship between anomaly scores and clinical annotations.

external dataset was collected using GE MUSE ECG systems, whereas PTB-XL was acquired using Schiller ECG systems. Population characteristics also differ between the two datasets. Despite these differences, the proposed method maintains good performance, demonstrating its robustness across datasets.

## References

1. Jiang A, Huang C, Cao Q, Wu S, Zeng Z, Chen K, et al. Multi-scale cross-restoration framework for electrocardiogram anomaly detection. In: International Conference on Medical Image Computing and Computer-Assisted Intervention. Springer; 2023. p. 87–97.

2. Wagner P, Strodthoff N, Bousseljot RD, Kreiseler D, Lunze FI, Samek W, et al. PTB-XL, a large publicly available electrocardiography dataset. Scientific Data. 2020;7(1):1–15.

3. Venkatesan C, Karthigaikumar P, Paul A, Satheeskumaran S, Kumar R. ECG signal preprocessing and SVM classifier-based abnormality detection in remote healthcare applications. IEEE Access. 2018;6:9767–9773.

4. Makowski D, Pham T, Lau ZJ, Brammer JC, Lespinasse F, Pham H, et al. NeuroKit2: A Python toolbox for neurophysiological signal processing. Behavior research methods. 2021;53(4):1689–1696.

5. Zheng J, Guo H, Chu H. A large scale 12-lead electrocardiogram database for arrhythmia study. PhysioNet. 2022;doi:10.13026/wgex-er52.
